# Impact of frailty status on the effect of a multidomain lifestyle intervention on cognition

**DOI:** 10.1093/ageing/afaf041

**Published:** 2025-02-26

**Authors:** Johanna Pöyhönen, Jenni Lehtisalo, Hanna-Maria Roitto, Esko Levälahti, Timo Strandberg, Miia Kivipelto, Jenni Kulmala, Riitta Antikainen, Hilkka Soininen, Jaakko Tuomilehto, Tiina Laatikainen, Tiia Ngandu

**Affiliations:** Faculty of Medicine, Clinicum, University of Helsinki, Helsinki, Finland; Department of Public Health, Finnish Institute for Health and Welfare, Helsinki, Finland; Department of Public Health, Finnish Institute for Health and Welfare, Helsinki, Finland; Institute of Clinical Medicine, Neurology, University of Eastern Finland, Kuopio, Finland; Faculty of Medicine, Clinicum, University of Helsinki, Helsinki, Finland; Department of Public Health, Finnish Institute for Health and Welfare, Helsinki, Finland; Department of Geriatrics, HUS Helsinki University Hospital, Helsinki, Finland; Department of Public Health, Finnish Institute for Health and Welfare, Helsinki, Finland; Faculty of Medicine, Clinicum, University of Helsinki, Helsinki, Finland; Department of Geriatrics, HUS Helsinki University Hospital, Helsinki, Finland; Center of Life Course Health Research, University of Oulu, Oulu, Finland; Department of Public Health, Finnish Institute for Health and Welfare, Helsinki, Finland; Division of Clinical Geriatrics, Center for Alzheimer Research, Care Sciences and Society (NVS), Karolinska Institutet, Stockholm, Sweden; Ageing Epidemiology Research Unit (AGE), School of Public Health, Imperial College London, London, United Kingdom of Great Britain and Northern Ireland; Theme Inflammation and Aging, Karolinska University Hospital, Stockholm, Sweden; Department of Public Health, Finnish Institute for Health and Welfare, Helsinki, Finland; Division of Clinical Geriatrics, Center for Alzheimer Research, Care Sciences and Society (NVS), Karolinska Institutet, Stockholm, Sweden; Faculty of Social Sciences (Health Sciences) and Gerontology Research Center (GEREC), Tampere University, Tampere, Finland; Center of Life Course Health Research, University of Oulu, Oulu, Finland; Medical Research Center, Oulu University Hospital, Oulu, Finland; Institute of Clinical Medicine, Neurology, University of Eastern Finland, Kuopio, Finland; Department of Neurology, Kuopio University Hospital, Neurocenter, Kuopio, Finland; Department of Public Health, Finnish Institute for Health and Welfare, Helsinki, Finland; National School of Public Health, Madrid, Spain; South Otsrobothnia Central Hospital, Seinäjoki, Finland; Department of Public Health, University of Helsinki, Helsinki, Finland; Department of Public Health, Finnish Institute for Health and Welfare, Helsinki, Finland; Institute of Public Health and Clinical Nutrition, University of Eastern Finland, Kuopio, Finland; Department of Public Health, Finnish Institute for Health and Welfare, Helsinki, Finland; Division of Clinical Geriatrics, Center for Alzheimer Research, Care Sciences and Society (NVS), Karolinska Institutet, Stockholm, Sweden

**Keywords:** frailty, prefrailty, cognition, lifestyle intervention, older people

## Abstract

**Background:**

Frailty often precedes and co-occurs with dementia. A multidomain lifestyle intervention has shown favourable effects on cognition. We aimed to investigate if frailty status modifies this intervention effect.

**Methods:**

The Finnish Geriatric Intervention Study to Prevent Cognitive Impairment and Disability (FINGER) recruited 1259 participants aged 60–77 years who were at risk of dementia. They were randomised to receive a multidomain intervention (diet, exercise, cognitive training and vascular risk monitoring) or regular health advice for two years. The outcome was a change in cognition (neuropsychological test battery composite score). Frailty and prefrailty were defined according to the Fried phenotype. Mixed models were used to investigate if frailty status at baseline modified the intervention effect on cognition.

**Results:**

Frailty status (prefrail/frail *n* = 520, robust *n* = 625) at baseline did not modify the effect of intervention on global cognition during the 2-year follow-up (*P*-value for frailty × intervention × time interaction > .05). Concerning cognitive subdomains, similar results were found. Among prefrail/frail persons, within-group analyses suggested a beneficial intervention effect on executive function and processing speed and also on global cognition when frail participants (*n* = 15) were excluded from the analyses. Being prefrail/frail was related to less improvement in global cognition, memory and executive function domains compared with being robust when intervention was not taken into consideration.

**Conclusions:**

A multidomain intervention is likely to be beneficial to cognition regardless of frailty status. Prefrail participants seemed particularly responsive to preventive intervention. Thus, an optimal time for a multidomain lifestyle intervention may be at the prefrailty stage.

## Key Points

- Frailty status at baseline did not modify the beneficial effect of a multidomain lifestyle intervention on cognition.
- Among prefrail participants, the beneficial effect was seen on executive function, processing speed and global cognition.
- Participants with stronger grip strength showed more improvement in global cognition than those with weaker grip strength.
- People with prefrailty could be an optimal target group for lifestyle intervention.

## Introduction

Healthy and independent ageing depends on both physical and cognitive abilities. Dementia currently affects >55 million people worldwide and is estimated to affect >150 million in 2050 [1]. Dementia is a complex and multifactorial syndrome with several potentially modifiable risk factors. A multidomain lifestyle intervention has been shown to be effective in preventing cognitive decline [2].

Frailty, another major geriatric syndrome, characterised by reduced resistance to various stressors but differentiated from disability, often precedes and co-occurs with dementia, which also has similar risk factors [3]. Frailty can be defined using a phenotype based on five characteristics [4]. Other commonly used frailty definitions include the frailty index (FI) [5], the Morley method [6] and various clinical frailty scales [7]. Due to the lack of unequivocal consensus regarding the definition of frailty, its prevalence in recent articles varies a lot; one systematic review has estimated it to be around 10% in community-dwelling older adults and increasing with age [8].

Several mechanisms of physical frailty (e.g. chronic systemic inflammation, oxidative stress, impaired hypothalamic–pituitary axis response, endocrine dysfunction and mitochondrial dysfunction) leading to imbalanced homeostasis have been suggested [9, 10]. This failing of multiple physiological systems in phenotypic frailty reduces functional capacity and resilience to stressors and results in an increased risk of illness, disability and death [11]. Similar mechanisms have been associated with cognitive decline and dementia [9, 10, 12]. Older adults with dementia have a high prevalence of frailty [13], and, while frailty predicts dementia [3, 14], cognitive impairment also predicts frailty [15].

The Finnish Geriatric Intervention Study to Prevent Cognitive Impairment and Disability (FINGER) was the first large randomised controlled trial showing that cognitive decline can be prevented by a 2-year multidomain lifestyle intervention including diet, exercise, cognitive training and vascular risk monitoring [2]. The trial has also shown that multidomain intervention is beneficial for physical function [16], quality of life [17] and incidence of chronic diseases [18]. A dementia risk score [19] used for selecting participants in the FINGER study includes midlife factors that have also been associated with frailty [20], indicating that the population could also be at risk for frailty. Thus, the FINGER trial provides a good opportunity for studying frailty and its interaction with cognition. To our knowledge, only a few studies have investigated frailty as a modifier of the lifestyle intervention effect on cognition, and they have shown no modifying effect of frailty. Also, these studies had some limitations with a small sample size, the cohort not representing the general population and the intervention not being multidomain [21, 22]. Due to population ageing and limited resources, it would be important to identify individuals who would most likely benefit from lifestyle intervention. Thus, we aimed to investigate if baseline frailty status has an impact on the effect of the multidomain lifestyle intervention on cognition in people at risk of dementia.

## Methods

### Study design and participants

We used data from the FINGER trial, which is a multidomain randomised controlled lifestyle intervention trial [2]. The active 2-year intervention period was in 2009–14. The study recruited 1259 at-risk older Finnish adults from the general population aged 60–77 years from previous representative risk factor surveys (FINRISK and FIN-D2D) [23]. Participants were selected using the validated dementia risk score [19] to indicate an elevated risk for dementia (≥6 points), and their cognitive performance was at the mean level or slightly lower for their age. The recruitment was done in six centres in Finland. In this study, we included participants with baseline data for frailty, baseline data for the neuropsychological test battery (NTB) total *z* score and at least one follow-up cognition assessment at 12 or 24 months.

### Trial protocol

Participants were randomly assigned to either a multidomain intervention or control group (1: 1). The intervention had four main components: nutrition, exercise, cognitive and social activity (including a computer-based training programme) and monitoring of metabolic/vascular risk. The control group received regular health advice.

Participants underwent annual study visits with the study nurse for anthropometric and blood pressure measurements and blood sample collection (e.g. lipids, glucose) during the 2-year intervention. A trained psychologist conducted the NTB assessment annually during the active intervention period (years 0, 1 and 2) to evaluate the primary outcome of the trial. Physical performance [including the short physical performance battery (SPPB), grip strength] was evaluated by a physiotherapist at baseline and at 2 years. Questionnaires on health and activities of daily living [22] were completed at every visit. The detailed trial design is described elsewhere [23].

The FINGER study was approved by the Coordinating Ethics Committee of the Hospital District of Helsinki and Uusimaa (94/13/03/00/2009). The study was conducted according to the guidelines laid down in the Declaration of Helsinki, and written informed consent was obtained from all participants at the start of the trial and at follow-up visits. This trial is registered with ClinicalTrials.gov (no. NCT01041989).

For the present study, we used the baseline data on frailty status, age, sex, education, body mass index (BMI), disease count (from a medical history questionnaire filled in by a physician after the participant interview), the APOE genotype (dichotomised as Ɛ4 carrier vs noncarrier), NTB score (also at 12 and 24 months) and adherence to intervention during 2 years [24].

### Definition of phenotypic frailty

Phenotypic frailty at baseline was defined by the modified Fried definition [4], where one point is assigned to each component that fulfils the criteria. ‘Weight loss’ was assessed with self-reported loss of weight during the previous 12 months, and ≥4.5 kg or ≥ 5% decrease was considered as weight loss. ‘Weakness’ was assessed with hand-grip strength (maximum result of two left and two right results) measured with a hydraulic hand dynamometer with original cut-offs by Fried (men BMI ≤24: ≤29 kg, BMI 24.1–26: ≤30 kg, BMI 26.1–28: ≤30 kg, BMI >28: ≤32 kg; women BMI ≤23: ≤17 kg, BMI 23.1–26: ≤17.3 kg, BMI 26.1–29: ≤18 kg, BMI >29: ≤21 kg). ‘Exhaustion’ was defined based on a question about weakness or tiredness during the previous month, and ‘a lot’ or ‘very much’ was considered exhaustion. ‘Low physical activity’ was assessed with a question: ‘How often do you in your leisure time exercise for at least 20 minutes so that you are at least mildly out of breath and sweaty?’. Those reporting once a week or less were considered inactive. ‘Slowness’ was assessed with gait speed (best result out of two 4-m walks with normal walking speed) with original Fried cut-offs for 15 feet adjusted for 4 m (men ≤173 cm: ≥6.15 s, >173 cm: ≥5.26 s; women ≤159 cm: ≥6.15 s, >159 cm: ≥5.26 s). Total frailty points of 0 were classified as robust, 1–2 as prefrail and 3–5 as frail. If a participant got one point on at least one of the frailty components, even if there were data missing on some of the components, the participant was included in the analyses as being prefrail or frail. If the participant had one or more missing components, and they had zero points on the nonmissing components, they were excluded from the analyses (*n* = 58).

### Cognitive outcomes

The outcome was a change in overall cognitive performance measured with a total score of an extended version of the NTB [25]. The NTB total score represents a composite score consisting of results from 14 cognitive tests. Test results were calculated as standardised *z* scores, with higher scores demonstrating better performance. Other cognitive outcomes included NTB *z* scores for specific domains: executive function, processing speed and memory. The content of these tests has been described elsewhere [2]. Cognitive assessments were performed by psychologists at baseline, 12 and 24 months. Participants who dropped out were invited to the final assessment at 24 months.

### Statistical analyses

Continuous variables at baseline are presented as means ± standard deviations and categorical variables as frequencies (%). Baseline group comparisons were performed with Student’s *t*-test, Mann–Whitney *U* test or chi-square test as appropriate.

Due to the limited number of frail individuals, frailty status was dichotomised (robust vs prefrail/frail) and frailty components were dichotomous (yes/no). Linear mixed effects regression models with maximum likelihood estimation were used to analyse the change in cognitive scores (total *z* score and *z* score for domains) as a function of frailty status (robust vs prefrail/frail), time (continuous variable coded as 0 for baseline, 1 for 12-month visit and 2 for 24-month visit), randomisation group (intervention vs control) and their interactions (frailty status × time, frailty status × intervention, time × intervention and frailty status × time × intervention). The same analyses for the change in total NTB *z* score were conducted as a function of frailty components (weight loss, weakness, exhaustion, low physical activity and slowness), time and randomisation group and their interactions.

In addition to analysing cognitive performance as a continuous variable, the change in cognitive performance from baseline to 24 months was categorised into either no decline (improvement or staying the same) or decline. Logistic regression analyses were used to assess if frailty status modified the intervention effect on change in cognition. Odds ratios (ORs) and *P*-values for cognitive decline were analysed for intervention and control groups (intervention group as reference) within each subgroup of frailty. Also, the interaction of frailty status × intervention was included.

We performed sensitivity analyses adjusting the models with age, sex, education (years) and number of chronic diseases (missing values imputed with mean; *n* = 1 for education and *n* = 6 for chronic diseases).

*P*-values of <.05 were considered statistically significant, and results are reported with 95% confidence intervals (CIs). Statistical analyses were performed on SPSS 29.0 for Windows (SPSS Inc., Chicago, IL, USA).

## Results

### Participant characteristics

**Figure 1:**
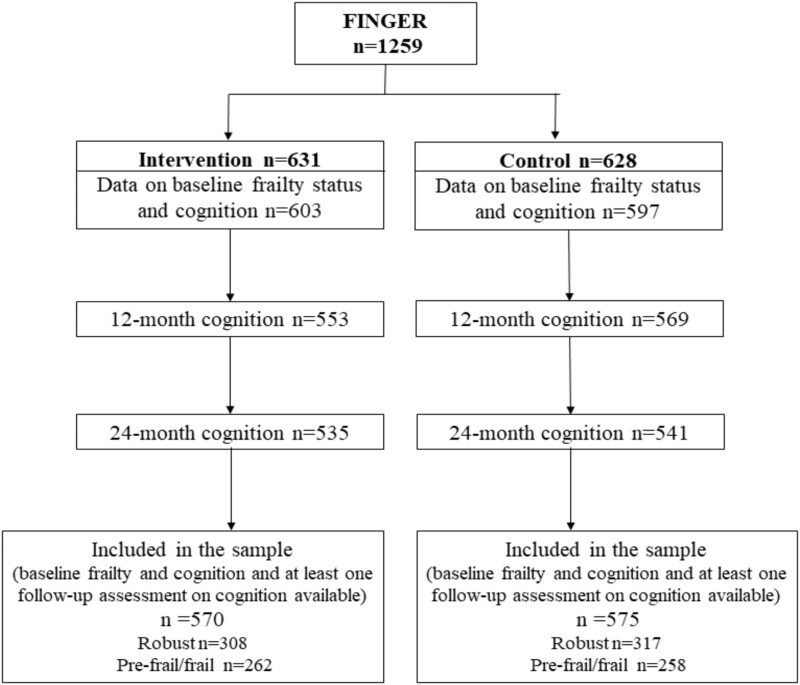
Flow chart. A total of 1145 of 1259 participants in the FINGER trial with reliable baseline data on frailty and cognition with at least one assessment of cognition at 12 or 24 months (mITT) were selected for the present study.

A total of 1145 (91%) participants out of 1259 were included in the analysis with data on frailty and global cognition at baseline and at least one cognition assessment at 12 or 24 months (modified intention-to-treat) (Figure 1). Of all participants, 625 (55%) were robust at baseline, 505 (44%) were prefrail and 15 (1%) were frail.

At baseline, the mean age of study participants was 69.3 years and 521 (45.5%) were female. People who were prefrail/frail had a higher BMI, more diseases, slower gait speed, weaker grip strength and lower processing speed domain compared with the robust group. Baseline frailty status was not associated with adherence to the intervention (Table 1). Comparisons between intervention and control groups within frailty subgroups are reported in Appendix Supplementary Table 1.

**Table 1:** Baseline characteristics of participants: comparison between robust and prefrail/frail

Characteristic	*N*	All patients (*n* = 1145)	Robust (*n* = 625)	Prefrail/frail (*n* = 520)	*P*-value
**Sociodemographics**					
Age (years)	1145	69.3 ± 4.7	69.3 ± 4.6	69.3 ± 4.7	.830
Female sex	1145	521 (45.5)	273 (43.7)	248 (47.7)	.175
Education (years)	1144	10.0 ± 3.4	10.1 ± 3.5	9.9 ± 3.3	.564
**Health factors**					
Body mass index (kg/m^2^)	1141	28.3 ± 4.8	27.5 ± 4.2	29.1 ± 5.2	**<.001**
Diseases (count)^a^	1139	2.5 ± 1.5	2.3 ± 1.5	2.7 ± 1.6	**<.001**
APOE Ɛ4 carrier (yes)^b^	1069	346 (32.4)	193 (32.9)	153 (31.7)	.662
**Frailty components** ^c^					
1. Weight loss (yes)	1142	102 (8.9)	NA	102 (19.7)	NA
2. Weakness: grip strength (yes)	1123	115 (10.2)	NA	115 (23.1)	NA
3. Exhaustion (yes)	1134	74 (6.5)	NA	74 (14.5)	NA
4. Low physical activity (yes)	1141	343 (30.1)	NA	343 (66.5)	NA
5. Slowness: gait speed (yes)	1125	22 (2.0)	NA	22 (4.4)	NA
Frailty points, total	1145	0.6 ± 0.7	NA	1.3 ± 0.5	NA
Grip strength (kg)	1127	34.50 ± 10.73	36.05 ± 9.83	32.56 ± 11.47	**<.001**
Gait speed (s)	1129	3.42 ± 0.84	3.25 ± 0.56	3.62 ± 1.06	**<.001**
**Cognition**					
NTB total score	1145	0.00 ± 0.58	0.01 ± 0.57	−0.01 ± 0.58	.509
NTB memory domain score	1145	0.00 ± 0.67	−0.02 ± 0.67	0.02 ± 0.67	.230
NTB executive function domain score	1144	0.00 ± 0.67	0.03 ± 0.67	−0.02 ± 0.67	.197
NTB processing speed domain score	1145	0.02 ± 0.81	0.07 ± 0.79	−0.05 ± 0.84	**.015**
**Adherence to intervention** ^**d**^	570				.492
1–6		203 (35.6)	105 (34.1)	98 (37.4)	
7		141 (24.7)	74 (24.0)	67 (25.6)	
8		226 (39.6)	129 (41.9)	97 (37.0)	

Data are numbers (percentages) of participants or means ± SD. Statistically significant *P*-values are bolded. Abbreviations: APOE, apolipoprotein E; NTB, neuropsychological test battery; NA, not applicable; SD, standard deviation.

^a^Mean count of 18 diagnoses; asked at baseline.

^b^Carrier of at least one APOE Ɛ4 allele vs non-carriers

^c^Frailty components: N (%) of participants scoring a point; point scoring explained in text; Frailty total points: mean points (5 max); pre-frailty 1–2 points, frailty 3 or more points.

^d^Intervention group only, categorised to 1–6, 7 and 8 (1 being the lowest adherence, 8 the highest) during 2-year intervention.

### Change in cognitive performance

The interaction between frailty status and time was significant for the NTB total score and memory and executive function domains, indicating less overall improvement (without considering the intervention effect) among the prefrail/frail group compared with the robust group (Table 2).

**Table 2:** The effect of baseline frailty status on cognition and the effect of lifestyle intervention on cognition modified by baseline frailty status

	*N*	Estimated change per year (95% CI)	*P*-value for interaction	Estimated difference between intervention and control subgroups per year (95% CI)	Estimated difference between frailty subgroups in intervention effect per year (95% CI)	*P*-value for interaction
**NTB total *Z* score**						
Robust	625	0.105 (0.092 to 0.119)	**.008**	0.014 (−0.014 to 0.041) *P* = .323	0.016 (−0.025 to 0.057)	.440
Prefrail/frail	520	0.078 (0.063 to 0.093)		0.030 (0.000 to 0.060) *P* = .053		
**Memory**						
Robust	625	0.187 (0.164 to 0.209)	**.023**	0.016 (−0.029 to 0.061) *P* = .494	−0.009 (−0.075 to 0.058)	.802
Prefrail/frail	520	0.148 (0.123 to 0.172)		0.007 (−0.042 to 0.057 *P* = .778		
**Executive function**						
Robust	624	0.051 (0.033 to 0.069)	**.033**	0.008 (−0.028 to 0.044) *P* = .675	0.044 (−0.010 to 0.097)	.107
Prefrail/frail	520	0.022 (0.002 to 0.042)		0.052 (0.012 to 0.091) ***P* = .011**		
**Processing speed**						
Robust	625	0.036 (0.018 to 0.055)	.963	0.020 (−0.018 to 0.057) *P* = .301	0.023 (−0.033 to 0.078)	.423
Prefrail/frail	520	0.037 (0.016 to 0.058)		0.042 (0.001 to 0.084) ***P* = .044**		

Mixed model analyses were used to assess estimated change in cognitive performance per year within frailty subgroups, difference in cognitive performance between frailty subgroups (frailty status × time interaction), difference in cognitive performance between intervention and control subgroups within frailty subgroups (positive value indicates that the effect is in favour of intervention group), and the difference in the intervention effect between frailty subgroups (frailty status × time × intervention interaction). Positive value indicates the effect of intervention being in favour of prefrail/frail subgroup. Statistically significant *P*-values are bolded. Abbreviations: CI, confidence interval; NTB, neuropsychological test battery.

**Figure 2:**
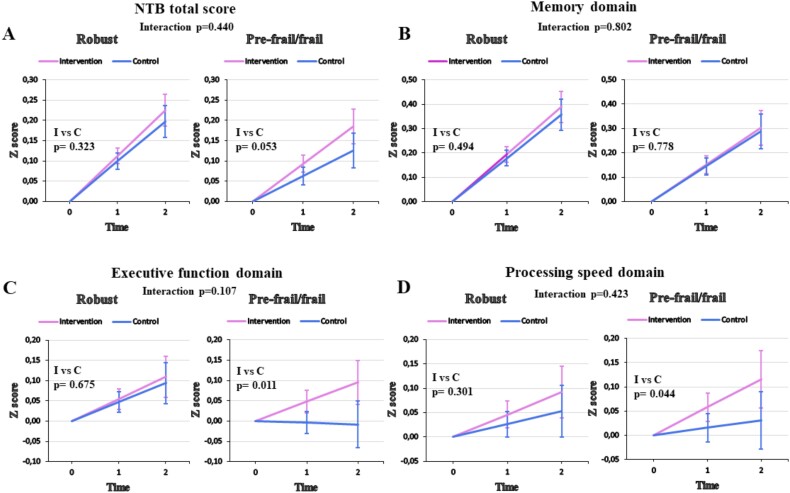
Intervention effect on cognitive performance (NTB total *z* score and domain *z* scores) modified by baseline frailty status (2 years). Estimated change in cognitive performance (total *z* score) in intervention and control groups within robust (A, left panel) and prefrail/frail (A, right panel) participants. *P*-values are for the difference between intervention and control groups within frailty subgroups (intervention × time) and the difference in intervention effect between frailty subgroups (frailty status × intervention × time). Error bars depict 95% confidence intervals. Same for the domain z scores (B–D). Abbreviations: NTB, Neuropsychological Test Battery; I, intervention; C, control.

Frailty status at baseline did not modify the intervention effect on global cognition (NTB total *z* score), with an estimated difference between robust and prefrail/frail groups in the intervention effect per year (frailty status × time × intervention) of 0.016 (95% CI −0.025 to 0.057), *P* = .440 (Table 2). For the NTB domains, there were no statistically significant three-way interactions between frailty status, time and intervention, but within-group analyses showed that the annual difference between intervention and control groups was statistically significant among the prefrail/frail group in executive function (estimate 0.052; 95% CI 0.012–0.091; *P* = .011) and processing speed (estimate 0.042; 95% CI 0.001–0.084; *P* = .044) (Figure 2).

We conducted the same analyses for robust vs prefrail people (excluding 15 frail participants) and found that even though the main result (the three-way interaction) stayed similar as with frail participants included, among prefrail people the intervention effect was even more evident in executive function (*P* = .007) and processing speed domains (*P* = .015), as well as global cognition (*P* = .018) (Supplementary Figure 1).

Concerning the individual components of frailty, participants with weaker grip strength showed less improvement in global cognition than those with stronger grip strength (grip strength × time *P* = .009, Table 3). No statistically significant three-way interactions (frailty component × time × intervention) on global cognition were observed. However, the intervention effect on executive function was superior in participants with low physical activity (*n* = 343) compared with active ones (*n* = 798) (physical activity × time × intervention), with an estimated difference per year of 0.060 (95% CI 0.002–0.119), *P* = .042, as well as in those with faster gait speed (*n* = 1103) compared with slower (*n* = 22) (slow gait speed × time × intervention), with an estimated difference per year of −0.212 [95% CI −0.413–(−0.012)], *P* = .038 (Supplementary Table 2).

**Table 3:** The effect of baseline frailty component on cognition and the effect of lifestyle intervention on cognition modified by baseline frailty component

		*N*	Estimated change per year (95% CI)	*P*-value for interaction	Estimated difference between intervention and control groups per year (95% CI)	Estimated difference between frailty component subgroups in intervention effect per year (95% CI)	*P*-value for interaction
**NTB total *Z* score**							
Frailty components							
1. Weight loss	No	1040	0.095 (0.084 to 0.105)	.413	0.020 (−0.001 to 0.042) *P* = .059	0.012 (−0.059 to 0.084)	.734
	Yes	102	0.080 (0.046 to 0.114)		0.033 (−0.035 to 0.101) *P* = .345		
2. Weakness: grip strength	No	1008	0.099 (0.088 to 0.109)	**.008**	0.022 (0.001 to 0.044) ***P* = .042**	−0.019 (−0.087 to 0.049)	.582
	Yes	115	0.053 (0.021 to 0.085)		0.003 (−0.061 to 0.067) *P* = 0.918		
3. Exhaustion	No	1060	0.096 (0.085 to 0.106)	.276	0.021 (0.000 to 0.042) ***P* = .046**	−0.032 (−0.116 to 0.051)	.449
	Yes	74	0.073 (0.032 to 0.113)		−0.011 (−0.092 to 0.070) *P* = .792		
4. Low physical activity	No	798	0.099 (0.086 to 0.111)	.102	0.020 (−0.004 to 0.044) *P* = .110	0.005 (−0.039 to 0.049)	.825
	Yes	343	0.080 (0.061 to 0.099)		0.025 (−0.012 to 0.062) *P* = .190		
5. Slowness: gait speed	No	1103	0.095 (0.084 to 0.105)	.585	0.023 (0.003 to 0.044) ***P* = .027**	−0.122 (−0.274 to 0.030)	.115
	Yes	22	0.073 (−0.002 to 0.149)		−0.099 (−0.250 to 0.052) *P* = .197		

Mixed model analyses were used to assess estimated change in cognitive performance per year within frailty component subgroups (0/1, frailty points explained in text), difference in cognitive performance between frailty component subgroups (frailty component × time interaction), difference in cognitive performance between intervention and control subgroups within frailty component subgroups (positive value indicates that the effect is in favour of intervention group) and the difference in the intervention effect between frailty component subgroups (frailty component × time × intervention interaction). Positive value indicates the effect being in favour of subgroup with 1 point. Statistically significant *P*-values are bolded. Abbreviations: CI, confidence interval; NTB, neuropsychological test battery.

The intervention effect on overall cognitive decline (0–24 months) (Supplementary Table 3) was not modified by frailty status in global cognition, memory or executive function domains. However, the risk of decline in processing speed was modified by frailty status, being greater among prefrail/frail individuals for those in the control than intervention group (OR 1.80) compared with robust individuals (OR 1.03, *P* = .027 for interaction).

In sensitivity analyses adjusted for age, sex, education and chronic diseases, the estimates were similar, and all statistically significant associations remained significant (results not shown).

## Discussion

This study found that phenotypic frailty status did not modify the overall beneficial effect of the multidomain lifestyle intervention on cognition among older adults at risk for dementia. However, prefrail participants seemed to be particularly responsive to the intervention effect on cognition. Regarding frailty components, physically inactive participants benefitted more than active ones in the executive function domain of cognition. Interestingly, when the outcome was categorised to assess the risk of cognitive decline during the 2-year intervention period, the intervention reduced the risk of decline in the processing speed domain among the prefrail/frail compared with the robust participants.

Only a few studies have investigated whether frailty modifies the effect of lifestyle intervention on cognition. Similar to our results, a study of adults with type 2 diabetes showed that frailty (defined by FI categorised into tertiles) had no impact on the effect of lifestyle intervention on cognition, but, as speculated by the authors, the cohort was quite young to undergo marked cognitive decline and lacked cognition assessment at baseline, and the participants had diabetes and overweight or obesity, thus not representing the general population [21]. Also, similar results were found by a study showing that a 12-week exercise training intervention benefits physical performance, cognition and quality of life in older adults, with equivalent results for frail (assessed with two out of three criteria: Fried phenotype, physical performance test and FI) and nonfrail participants [22]. However, the study cohort was underpowered to investigate interactions between frailty and intervention and the intervention period was short. Comparison with previous reports is difficult because definitions of frailty, inclusion criteria and interventions were different. Additionally, the latter study compared frail with nonfrail people, whereas our study population comprised mainly robust and prefrail individuals and only a few persons were categorised as frail. Therefore, results from our study add to the current knowledge that in community-dwelling older adults, a multimodal lifestyle intervention targeting several risk factors at the same time is beneficial for cognition, irrespective of baseline frailty status. Nevertheless, it is important to note that prefrail people received the most benefits from the lifestyle intervention.

There are studies suggesting that frailty affects executive function and processing speed more than memory [26, 27]. Our results do not fully support that, because less improvement in global cognition and memory domain was found among prefrail/frail participants in addition to executive function. Still, the prefrail/frail subgroup was especially responsive to intervention in these domains. Furthermore, in the frailty component analyses, low physical activity and in assessing cognitive decline risk during the 2-year intervention, frailty status, did modify the intervention effect in executive function and processing speed domains of cognition. Weak grip strength and slow gait speed are the components of frailty shown to be strongly associated with low cognitive functioning [28]. Our finding of weak grip strength as a predictor of worse cognitive improvement is in keeping with this.

Strengths of our study include a large study population, randomised trial design and carefully planned intervention with a long duration. The dementia risk score [19] used in participant selection includes many risk factors relevant to frailty, and the multidomain lifestyle intervention targeted several of these underlying mechanisms. However, there are some limitations to this study. The original number of study participants was calculated to have statistical power in comparing intervention and control groups. Thus, the possibility of limited statistical power should be considered when interpreting the nonsignificant three-way interaction results. Also, dividing participants into smaller subgroups may increase false-positive findings; thus, interpretation of positive within-group findings should be done with caution. Group sizes with low statistical power existed, especially in frailty component analyses where people with no frailty component comprised most of the participants, except in low physical activity. The data collection was not originally designed to carry out substudies, e.g. to capture effects of frailty, which may have led to some misclassification of frailty. We tried to stay with the original definition of the Fried phenotype as much as possible. However, weight loss was originally classified as involuntary, but that information was not available in this study. Additionally, weight loss was self-reported, creating further uncertainty. Low physical activity was assessed with a different method from the original definition, where it was calculated as kcal expended. Lastly, in our prefrail/frail subgroup, the number of frail individuals was too small to draw conclusions concerning frailty alone.

The clinical value of this study is that even though similar multidomain interventions can be implemented without initially screening for frailty, as also robust persons appear to benefit, the within-group findings indicate that prefrail individuals may benefit most from such intervention. Whether this beneficial effect is more pronounced among people with prefrailty should be further investigated in specifically designed studies. Interestingly, a recent study suggested that prefrailty may serve as an entry point into the process of disability, offering a possibility for early interventions [29]. Perhaps the optimal timing for a preventive intervention would be before becoming frail, thus helping us in targeting limited resources. It is also possible that people with frailty would need more intensive intervention or more support to benefit from lifestyle interventions.

## Conclusions

A multidomain lifestyle intervention, such as that used in the FINGER trial, is likely to be beneficial for cognition in both robust and phenotypic prefrail/frail participants. People with prefrailty might be particularly responsive to interventions, and this may be an optimal time for a lifestyle intervention.

## Supplementary Material

aa-24-1734-File002_afaf041

## Research Data Transparency and Availability:

The data presented in this article are not available because public deposition of the de-identified data is not possible due to legal and ethical reasons, and complete deidentification is not possible as this study is part of an ongoing trial. The participants gave informed consent, which includes data use only under a confidentiality agreement. The data contain sensitive information, and public data deposition may cause privacy concerns. Those fulfilling the requirements for viewing confidential data as required by Finnish law and the Finnish Institute for Health and Welfare are able to access the data after completion of material transfer agreement. Requests to access the data should be directed to kirjaamo@thl.fi.
