# Supplementary material for: Impact of frailty status on the effect of a multidomain lifestyle intervention on cognition
Source: Age Ageing. 2025 Feb 26;54(2):afaf041. doi: 10.1093/ageing/afaf041 (PMC11878527; doi:10.1093/ageing/afaf041)
Supplement: aa-24-1734-File002_afaf041 [file aa-24-1734-file002_afaf041.docx]

**Impact of frailty status on the effect of a multidomain lifestyle intervention on cognition**

**APPENDIX Content:**

**Supplementary Table 1**. Comparison of baseline characteristics between intervention and control groups in frailty subgroups.

**Supplementary Figure 1**. Intervention effect on cognitive performance (NTB total z score and domain z scores) modified by baseline pre-frailty. Frail participants (N=15) excluded from the analyses**.**

**Supplementary Table 2**. The effect of baseline frailty component on cognition (NTB domains) and the effect of lifestyle intervention on cognition (NTB domains) modified by baseline frailty component.

**Supplementary Table 3**. Risk of cognitive decline (intervention vs control) from baseline to 24 months in frailty subgroups and modified by frailty status.

| **Supplementary Table 1.** **Comparison of baseline characteristics between intervention and controls groups in frailty sub-groups** | | | | | | | |
| --- | --- | --- | --- | --- | --- | --- | --- |
| **Characteristic** |  | **Robust** | |  | **Pre-frail/frail** | |  |
|  | **N** | **Intervention** (n=308) | **Control** (n=317) | ***P*-value** | **Intervention** (n=262) | **Control** (n=258) | ***P*-value** |
| **Sosiodemographics** |  |  |  |  |  |  |  |
| Age (years) | 1145 | 69.6±4.6 | 68.9±4.7 | **0.048** | 69.2±4.7 | 69.4±4.8 | 0.742 |
| Female sex | 1145 | 134 (43.5) | 139 (43.8) | 0.931 | 120 (45.8) | 128 (49.6) | 0.384 |
| Education (years) | 1144 | 10.0±3.6 | 10.1±3.5 | 0.298 | 9.9±3.2 | 9.9±3.4 | 0.408 |
| **Health factors** |  |  |  |  |  |  |  |
| Body mass index (kg/m²) | 1141 | 27.6±4.0 | 27.5±4.4 | 0.676 | 29.4±4.9 | 28.9±5.5 | 0.244 |
| Diseases (count)^a^ | 1139 | 2.2±1.5 | 2.3±1.4 | 0.266 | 2.8±1.6 | 2.6±1.6 | 0.113 |
| APOE Ɛ4 carrier (yes)^b^ | 1069 | 93 (32.6) | 100 (33.2) | 0.879 | 68 (28.3) | 85 (35.0) | 0.116 |
| **Frailty components**^c^ |  |  |  |  |  |  |  |
| 1. Weight loss (yes) | 1142 | NA | NA | NA | 55 (21.2) | 47 (18.3) | 0.413 |
| 2. Weakness: grip strength (yes) | 1123 | NA | NA | NA | 58 (23.0) | 57 (23.2) | 0.967 |
| 3. Exhaustion (yes) | 1134 | NA | NA | NA | 40 (15.6) | 34 (13.5) | 0.507 |
| 4. Low physical activity (yes) | 1141 | NA | NA | NA | 175 (67.6) | 168 (65.4) | 0.597 |
| 5. Slowness: gait speed (yes) | 1125 | NA | NA | NA | 11 (4.3) | 11 (4.5) | 0.939 |
| Frailty points total | 1145 | NA | NA | NA | 1.3±0.5 | 1.2±0.5 | 0.151 |
| Grip strength (kg) | 1127 | 35.64±9.60 | 36.44±10.05 | 0.377 | 32.77±11.40 | 32.36±11.55 | 0.427 |
| Gait speed (s) | 1129 | 3.25±0.57 | 3.26±0.55 | 0.565 | 3.62±1.15 | 3.62±0.95 | 0.568 |
| **Cognition** |  |  |  |  |  |  |  |
| NTB total score | 1145 | -0.04±0.55 | 0.07±0.59 | **0.016** | -0.01±0.57 | -0.01±0.59 | 0.999 |
| NTB memory domain score | 1145 | -0.08±0.66 | 0.03±0.68 | **0.030** | 0.03±0.70 | 0.02±0.64 | 0.907 |
| NTB executive function domain score | 1144 | -0.03±0.68 | 0.08±0.66 | **0.033** | -0.02±0.63 | -0.02±0.72 | 0.983 |
| NTB processing speed domain score | 1145 | 0.02±0.77 | 0.12±0.80 | 0.097 | -0.05±0.80 | -0.04±0.88 | 0.918 |
| Data are numbers (percentages) of participants or means ± SD. ^a^ Mean count of 18 diagnoses; asked at baseline. ^b^ Carrier of at least one APOE Ɛ4 allele vs non-carriers ^c^ Frailty components: N (%) of participants scoring a point; point scoring explained in text; Frailty total points: mean points (5 max); pre-frailty 1–2 points, frailty 3 or more points. *Abbreviations: APOE = apolipoprotein E; NTB= Neuropsychological test battery; NA= not applicable; SD=standard deviation* | | | | | | | |

**Supplementary Figure 1.**


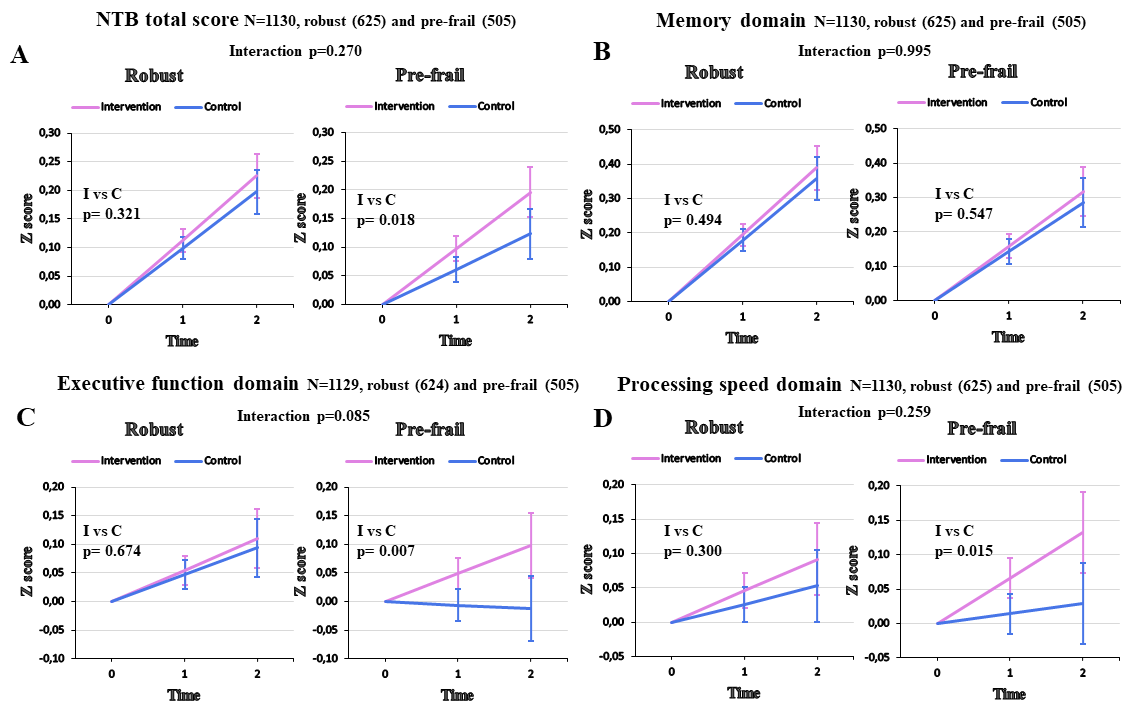


**Supplementary Figure** **1. Intervention effect on cognitive performance (NTB total z score and domain z scores) modified by baseline pre-frailty. Frail participants (N=15) excluded from the analyses.** Estimated change in cognitive performance (total z score) in intervention and control groups within robust (**A, left panel**) and pre-frail (**A, right panel**) participants. *P*-values are for the difference between intervention and control groups within frailty subgroups (intervention × time) and the difference in intervention effect between frailty subgroups (frailty status × intervention × time). Error bars depict 95% confidence intervals. Same for domain z scores (**B, C, D)**. *Abbreviations: NTB =Neuropsychological test battery, I=Intervention, C=Control.*

**Alt text:** Graphics on the results of statistical analyses comparing intervention effect on cognition within and between robust and pre-frail groups with subfigures labeled from A to D concerning global cognition and cognition subdomains.

| **Supplementary Table 2. The effect of baseline frailty component on cognition (domains) and the effect of lifestyle intervention on cognition (domains) modified by baseline frailty component** | | | | | | | |
| --- | --- | --- | --- | --- | --- | --- | --- |
|  |  | **N** | **Estimated change per year (95% CI)** | **P-value for interaction** | **Estimated difference between intervention and control groups per year (95% CI)** | **Estimated difference between frailty component subgroups in intervention effect per year (95% CI)** | ***P*-value for interaction** |
| **Memory** |  |  |  |  |  |  |  |
| Frailty components |  |  |  |  |  |  |  |
| 1. Weight loss | no | 1040 | 0.170 (0.153–0.188) | 0.882 | 0.012 (-0.023–0.047) p=0.514 | 0.003 (-0.114–0.120) | 0.960 |
|  | yes | 102 | 0.163 (0.107–0.219) |  | 0.015 (-0.097–0.126) p=0.797 |  |  |
| 2. Weakness (grip strength) | no | 1008 | 0.177 (0.160–0.195) | **0.005** | 0.013 (-0.022–0.048) p=0.476 | -0.008 (-0.119–0.103) | 0.891 |
|  | yes | 115 | 0.097 (0.045–0.150) |  | 0.005 (-0.100–0.110) p=0.925 |  |  |
| 3. Exhaustion | no | 1060 | 0.172 (0.155–0.189) | 0.739 | 0.009 (-0.025–0.044) p=0.592 | -0.19 (-0.156–0.118) | 0.785 |
|  | yes | 74 | 0.160 (0.094–0.227) |  | -0.010 (-0.142–0.123) p=0.886 |  |  |
| 4. Low physical activity | no | 798 | 0.179 (0.159–0.199) | 0.091 | 0.026 (-0.014–0.066) p=0.205 | -0.046 (-0.119–0.027) | 0.215 |
|  | yes | 343 | 0.148 (0.117–0.178) |  | -0.020 (-0.081–0.041) p=0.513 |  |  |
| 5. Slowness (gait speed) | no | 1103 | 0.172 (0.155–0.189) | 0.191 | 0.015 (-0.019–0.049) p=0.393 | -0.084 (-0.334–0.166) | 0.511 |
|  | yes | 22 | 0.088 (-0.036–0.212) |  | -0.069 (-0.317–0.179) p=0.585 |  |  |
| **Executive function** |  |  |  |  |  |  |  |
| Frailty components |  |  |  |  |  |  |  |
| 1. Weight loss | no | 1040 | 0.040 (0.026–0.054) | 0.422 | 0.025 (-0.003–0.053) p=0.082 | 0.041 (-0.053–0.135) | 0.388 |
|  | yes | 102 | 0.021 (-0.024–0.065) |  | 0.066 (-0.024–0.156) p=0.148 |  |  |
| 2. Weakness (grip strength) | no | 1008 | 0.043 (0.029–0.057) | 0.135 | 0.026 (-0.002–0.054) p=0.073 | -0.004 (-0.094–0.085) | 0.928 |
|  | yes | 115 | 0.009 (-0.034–0.051) |  | 0.022 (-0.063–0.107) p=0.614 |  |  |
| 3. Exhaustion | no | 1060 | 0.042 (0.029–0.056) | **0.048** | 0.030 (0.002–0.057) **p=0.034** | -0.045 (-0.154–0.065) | 0.423 |
|  | yes | 74 | -0.013 (-0.066–0.040) |  | -0.015 (-0.121–0.091) p=0.782 |  |  |
| 4. Low physical activity | no | 798 | 0.043 (0.027–0.059) | 0.201 | 0.010 (-0.022–0.042) p=0.535 | 0.060 (0.002–0.119) | **0.042** |
|  | yes | 343 | 0.024 (0.000–0.049) |  | 0.071 (0.022–0.119) **p=0.005** |  |  |
| 5. Slowness (gait speed) | no | 1103 | 0.039 (0.025–0.052) | 0.758 | 0.029 (0.002–0.056) **p=0.034** | -0.212 (-0.413– (-0.0.12)) | **0.038** |
|  | yes | 22 | 0.054 (-0.045–0.154) |  | -0.183 (-0.382–0.015) p=0.071 |  |  |
| **Processing speed** |  |  |  |  |  |  |  |
| Frailty components |  |  |  |  |  |  |  |
| 1. Weight loss | no | 1040 | 0.038 (0.023–0.052) | 0.521 | 0.032 (0.003–0.061) **p=0.030** | -0.022 (-0.119–0.076) | 0.662 |
|  | yes | 102 | 0.022 (-0.024–0.068) |  | 0.011 (-0.082–0.103) p=0.824 |  |  |
| 2. Weakness (grip strength) | no | 1008 | 0.038 (0.023–0.052) | 0.915 | 0.035 (0.005–0.064) **p=0.021** | -0.039 (-0.132–0.054) | 0.412 |
|  | yes | 115 | 0.040 (-0.004–0.084) |  | -0.004 (-0.092–0.084) p=0.926 |  |  |
| 3. Exhaustion | no | 1060 | 0.036 (0.022–0.051) | 0.886 | 0.034 (0.006–0.063) **p=0.019** | -0.066 (-0.180–0.048) | 0.257 |
|  | yes | 74 | 0.040 (-0.015–0.096) |  | -0.032 (-0.142–0.079) p=0.574 |  |  |
| 4. Low physical activity | no | 798 | 0.034 (0.018–0.051) | 0.675 | 0.023 (-0.010–0.056) p=0.170 | 0.023 (-0.037–0.084) | 0.455 |
|  | yes | 343 | 0.041 (0.016–0.066) |  | 0.046 (-0.004–0.097) p=0.074 |  |  |
| 5. Slowness (gait speed) | no | 1103 | 0.037 (0.023–0.051) | 0.487 | 0.032 (0.004–0.060) **p=0.025** | -0.076 (-0.285–0.132) | 0.472 |
|  | yes | 22 | 0.074 (-0.029–0.177) |  | -0.044 (-0.251–0.162) p=0.674 |  |  |
| Mixed model analyses were used to assess estimated change in cognitive performance (NTB z score for domains) per year within frailty component subgroups (0/1, frailty points explained in text), difference in cognitive performance between frailty component subgroups (frailty component × time interaction), difference in cognitive performance between intervention and control subgroups within frailty component subgroups (positive value indicates that the effect is in favour of intervention group), and the difference in the intervention effect between frailty component subgroups (frailty component ×time × intervention interaction). Positive value indicates the effect being in favour of 1 point subgroup. *Abbreviations: CI = confidence interval, NTB = Neuropsychological test battery* | | | | | | | |

| **Supplementary Table 3. Risk of cognitive decline (intervention vs control) from baseline to 24 months in frailty subgroups and modified by frailty status** | | | | | |
| --- | --- | --- | --- | --- | --- |
|  | **N** | **Odds ratio (95% CI)** | | **P-value** | ***P*-value for interaction** |
|  |  | **Intervention** | **Control** |  |  |
| **NTB total Z score** |  |  |  |  |  |
| Robust | 593 | 1 | 1.14 (0.79–1.66) | 0.486 | 0.301 |
| Pre-frail/frail | 483 | 1 | 1.52 (1.03–2.25) | **0.036** |  |
| **Memory** |  |  |  |  |  |
| Robust | 593 | 1 | 1.12 (0.77–1.62) | 0.556 | 0.588 |
| Pre-frail/frail | 484 | 1 | 1.30 (0.88–1.92) | 0.194 |  |
| **Executive function** |  |  |  |  |  |
| Robust | 590 | 1 | 1.15 (0.82–1.60) | 0.417 | 0.428 |
| Pre-frail/frail | 480 | 1 | 1.40 (0.97–2.01) | 0.070 |  |
| **Processing speed** |  |  |  |  |  |
| Robust | 592 | 1 | 1.03 (0.75–1.43) | 0.841 | **0.027** |
| Pre-frail/frail | 482 | 1 | 1.80 (1.25–2.59) | **0.002** |  |
| Cognitive decline was defined as decrease in NTB total score (overall decline) and NTB domain  scores (decline per domain) between the assessments at baseline and at 24 months. Logistic regression analyses were  used to assess risk of cognitive decline in the control group compared with the intervention group within frailty subgroups, and the difference in the intervention effect between frailty subgroups (frailty status × intervention interaction). Analyses are based on all participants with data available at both baseline and 24 months. *Abbreviations: CI = confidence interval, NTB = Neuropsychological test battery*. | | | | | |
